# Supplementary material for: Cloud BioLinux: pre-configured and on-demand bioinformatics computing for the genomics community
Source: BMC Bioinformatics. 2012 Mar 19;13:42. doi: 10.1186/1471-2105-13-42 (PMC3372431; doi:10.1186/1471-2105-13-42)
Supplement: Additional file 1 — Supplementary 1 Cloud BioLinux software documentation in the form of a mini, self-contained website. Users need to download and uncompress the .zip file, and open through a web browser the "index.html" file available on the main directory. (ZIP 1823 kb). [file 1471-2105-13-42-S1.ZIP › Cloud-BioLinux-Package-Documentation/docs/boxshade.html]

Bio-Linux Software Documentation Pages

Back to search form

## boxshade

|  |  |
| --- | --- |
| Name | boxshade |
| Description | Boxshade is a program for creating visually pleasing images of multiple alignments of protein or DNA sequences.  Boxshade requires a file containing the aligned sequences in msf and aln formats.  Various kinds of shading can be applied to identical/similar residues. Output is written to screen or to a file in the following formats: ANSI/VT100, PS/EPS, RTF, HPGL, ReGIS, LJ250-printer, ASCII, xFIG, PICT, or HTML.  Boxshade has a maximum limit of150 sequences with up to 10000 elements each . The boxshade package for installation on Debian or Ubuntu systems is prepared by Debian-med. It can be installed on Bio-Linux by typing: `sudo apt-get install boxshade` For further information about boxshade, please refer to the < href="http://www.ch.embnet.org/software/BOX\_faq.html">remote documentation. |
| Homepage | http://www.ch.embnet.org/software/BOX\_form.html |
| Remote Documentation | http://www.ch.embnet.org/software/BOX\_faq.html |
